# Post-mortem Interval Estimation Through Entomology Using Calliphorid Flies: A Case Series

**DOI:** 10.7759/cureus.85875

**Published:** 2025-06-12

**Authors:** Arwinder Singh, Yashpal. S, Ashish R Bhute, Amit Jangid

**Affiliations:** 1 Forensic Medicine and Toxicology, All India Institute of Medical Sciences, Rishikesh, IND

**Keywords:** accumulated degree hours (adh), calliphorid fly, decomposition, forensic entomology, forensic medicine, insect colonization, maggot activity, post-mortem interval (pmi)

## Abstract

Post-mortem interval (PMI) estimation is a critical component of death investigations but becomes particularly challenging in cases involving advanced decomposition, where traditional methods may prove less effective. The combined effects of severe tissue damage, environmental conditions, and interactions with carrion feeders complicate the PMI assessment. Forensic entomology has been utilized to address these challenges by focusing on insect colonization patterns and life cycle analysis. This article emphasizes the value of forensic entomology as an essential tool for estimating the PMI in complex cases involving extensive decomposition, offering a reliable complement to conventional forensic techniques. In addition, this article outlines a straightforward approach for deriving PMI estimates from available data when direct access to a forensic entomologist is not feasible.

## Introduction

Estimating the post-mortem interval (PMI) is a fundamental objective of an autopsy using various methods, including observations of biochemical changes in body fluids, rigor mortis, livor mortis, and algor mortis [1]. However, the accuracy of these methods is often limited by inter-individual variability, environmental factors, and potential post-mortem manipulation of the body by offenders or during recovery. The complexity of PMI estimation increases significantly in cases of advanced decomposition owing to extensive tissue damage [2].

Given these complexities, the estimation of PMI requires forensic entomology, which is the study of insects to aid legal proceedings. This report highlights how examining insect species present (Calliphorid fly in our cases), their developmental stages, and succession patterns of colonization can be leveraged to construct a reliable PMI estimate [3]. These findings demonstrate the utility of forensic entomology as an essential tool for PMI estimation in cases where traditional methods are compromised by decomposition.

Calliphorid fly (blowflies)

Calliphorid flies (blowflies) are ectothermic insects of paramount forensic significance, primarily utilized for minimum post-mortem interval (mPMI) estimation. Adult females exhibit rapid oviposition on carrion, typically within minutes to hours post-mortem, attracted by putrefactive volatile organic compounds. Larval instars, known as maggots, then commence necrophagous feeding and undergo predictable, temperature-dependent development. Forensic entomologists determine the age of the oldest cohort of larvae or pupae based on species-specific growth curves, often employing accumulated degree hours/days (ADH/ADD) models. This entomological evidence provides a robust mPMI, particularly in cases where traditional indicators are unreliable [2-4].

In this case series, we have used the ADH/ADD model, which uses the growing degree day (GDD) value and ADH to calculate/estimate the mPMI. GDD quantifies how much a daily mean temperature exceeds the lower threshold temperature, which is crucial for understanding biological development. It is calculated simply as: GDD=average daily temperature-threshold temperature. Building on this, ADH is a refined unit representing the total thermal summation an organism experiences above its developmental threshold. It is derived by multiplying the GDDs by the time in hours: ADH=GDD×time (in hours). These metrics are fundamental in fields like forensic entomology for predicting insect development and are used in cases in this report [2-5].

Methodology 

The material required for PMI estimation using entomology includes climatological and temperature data (the location where the body was found), maggots or eggs, rearing containers (small plastic containers), rearing media (animal tissue), 80% ethyl or isopropyl alcohol, and measuring scale [6].

The methodology for PMI estimation using entomology is a stepwise process that includes the preservation and rearing of larvae or eggs, measurement and identification of larval stages, species identification from reared adult flies, and calculation [7].

mPMI estimation is initiated by the meticulous collection of insect larvae (maggots) from decomposing remains, with a portion immediately preserved in 80% ethyl or isopropyl alcohol for subsequent morphological measurement and larval stage identification. Concurrently, a separate cohort of live maggots is reared under controlled laboratory conditions to observe their time to maturation into adult flies, enabling definitive species identification. We chose 28°C as the incubation temperature because it is within the established optimal range for the predictable development of specific insect species, such as Lucilia sericata, as widely documented in forensic entomology literature. This temperature facilitates consistent and robust larval growth, which is crucial for accurate PMI estimation [8]. For the culture medium, we utilized 5 grams of pork meat per container, due to the recognized use of pig corpses as an analog to human cadavers in earlier forensic taphonomy research [9]. This observed developmental time facilitates the calculation of ADH consumed during the rearing phase. Crucially, species-specific reference ADH data for complete development to adulthood is then extracted from established literature. By subtracting the laboratory-accumulated ADH from the total reference ADH, the ADH accumulated before maggot collection is determined. This pre-collection ADH is then correlated with historical climatological and temperature data from the body recovery site to retrospectively calculate the mPMI, pinpointing the earliest possible time of insect colonization [6].

## Case presentation

Three cases were received in the mortuary (for post-mortem examination) in an advanced state of decomposition, with maggots all over the body. All the bodies were found in the external environment in different seasons. Different stages of larvae of Calliphorid fly were found on the bodies, which led to the estimation of the PMI of the respective bodies. 

Case 1

**Figure 1:**
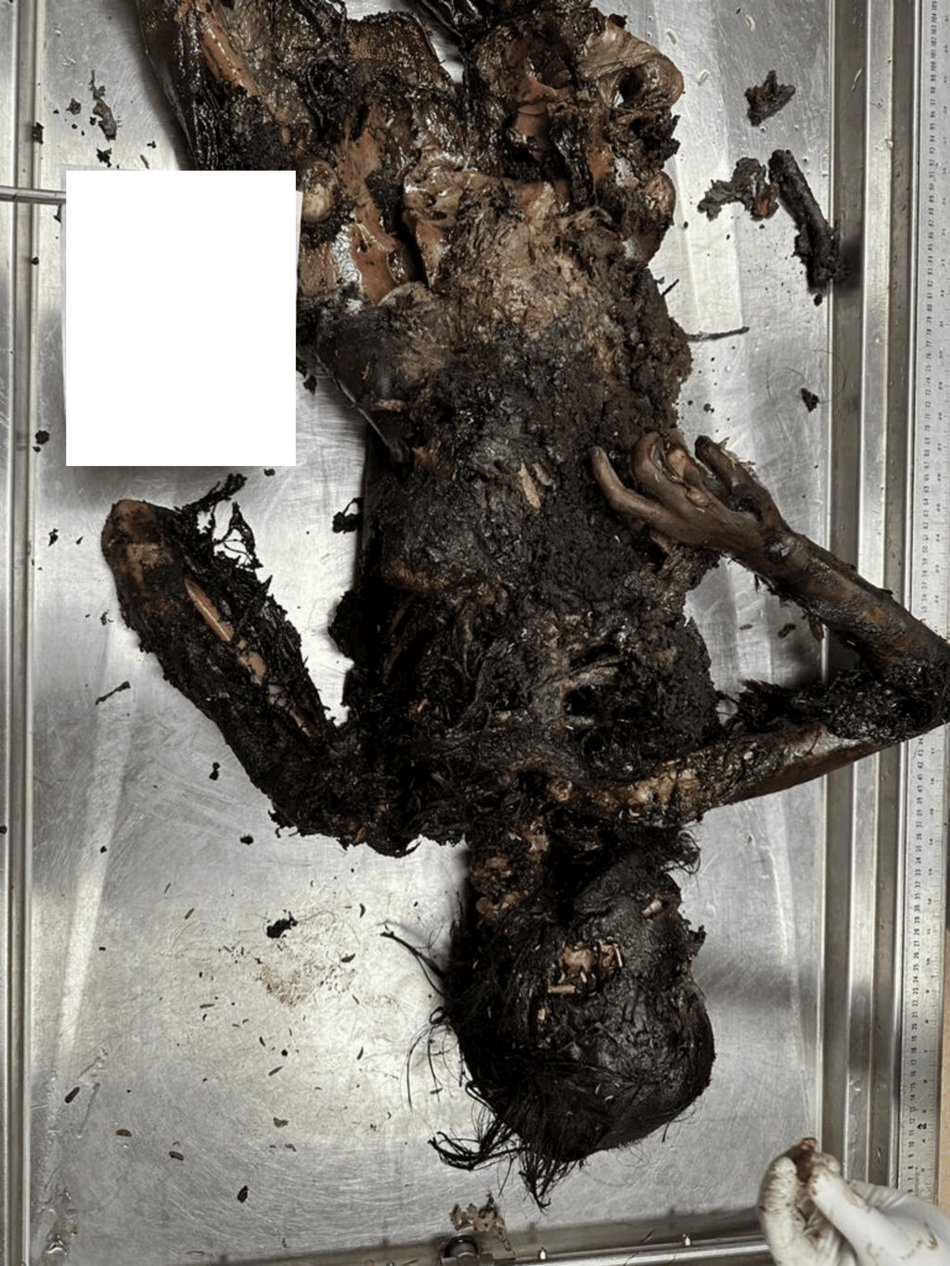
State of body received (Case 1) Advanced decomposition of the body in Case 1, showing extensive maggot and animal activity, with loss of skin and soft tissues due to self-immolation.

**Figure 2:**
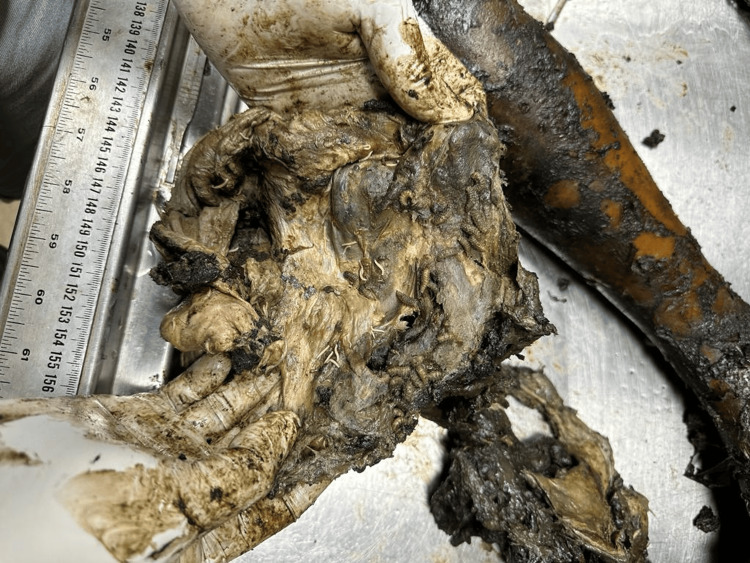
Maggots (Case 1) Close-up view of maggots colonizing the decomposed body in Case 1, indicating active insect involvement in tissue degradation.

**Figure 3:**
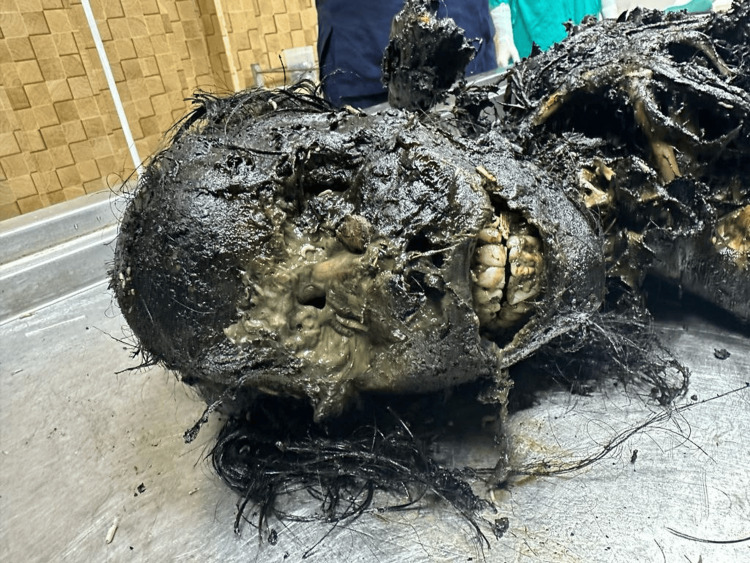
Unrecognizable face (Case 1) Unrecognizable facial features in Case 1 due to severe putrefaction and larval infestation.

A 21-year-old female's body was recovered from a forested area in the winter season, 12 days after being reported missing. The history suggests death by self-immolation. The body showed advanced decomposition, with most of the skin and soft tissue absent due to extensive maggot and animal activity (Figure 1). The face was unrecognizable due to severe putrefaction (Figure 2). Larval development ranged from <2 mm maggots to post-feeding stages, indicating sustained insect colonization (Figure 3). Internally, thoracic and abdominal organs had turned into a blackish mass, and pelvic organs were entirely absent, likely due to insect scavenging. Animal predation was evident.

GDD value and ADH from the day of missing to the day of recovery of the body are shown in Table 1.

**Table 1:** Case 1: GDD and ADH from the day of missing to the day of recovery of the body Case 1: Daily average temperature, threshold temperature, GDD, and ADH from the day of disappearance to body recovery. ADH: accumulated degree hours; GDD: growing degree day value

Day	Avg temp	Threshold temp	GDD value (avg temp-threshold temp)	ADH (GDDx24 hrs)	Adding ADH from the day of recovery to the day of missing
D-0	17	10	7	168	1816
D-1	17	10	7	168	1648
D-2	18	10	8	192	1480
D-3	18	10	8	192	1288
D-4	17	10	7	168	1096
D-5	16	10	6	144	928
D-6	16.5	10	6.5	156	784
D-7	14.5	10	4.5	108	628
D-8	14.5	10	4.5	108	520
D-9	14.5	10	4.5	108	412
D-10	15.5	10	5.5	132	304
D-11	14.5	10	4.5	108	172
D-12 (up to 4 PM)	14	10	4	64	64

**Figure 4:**
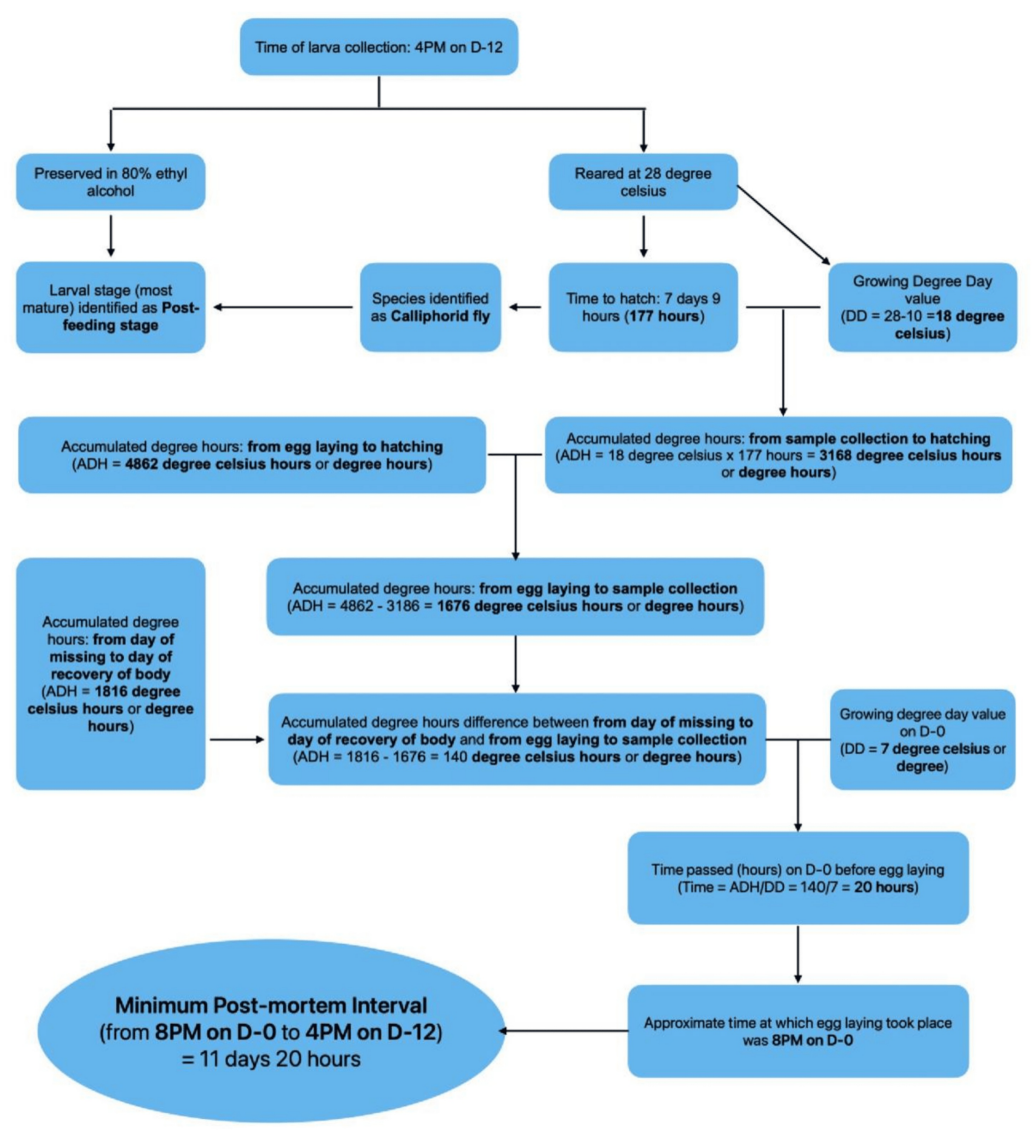
Flowchart: PMI estimation for Case 1 Flowchart illustrating the estimation of PMI for Case 1 using ADH. PMI: post-mortem interval; ADH: accumulated degree hours

mPMI estimated was about 11-12 days prior to post-mortem examination (Figure 4).

Case 2

**Figure 5:**
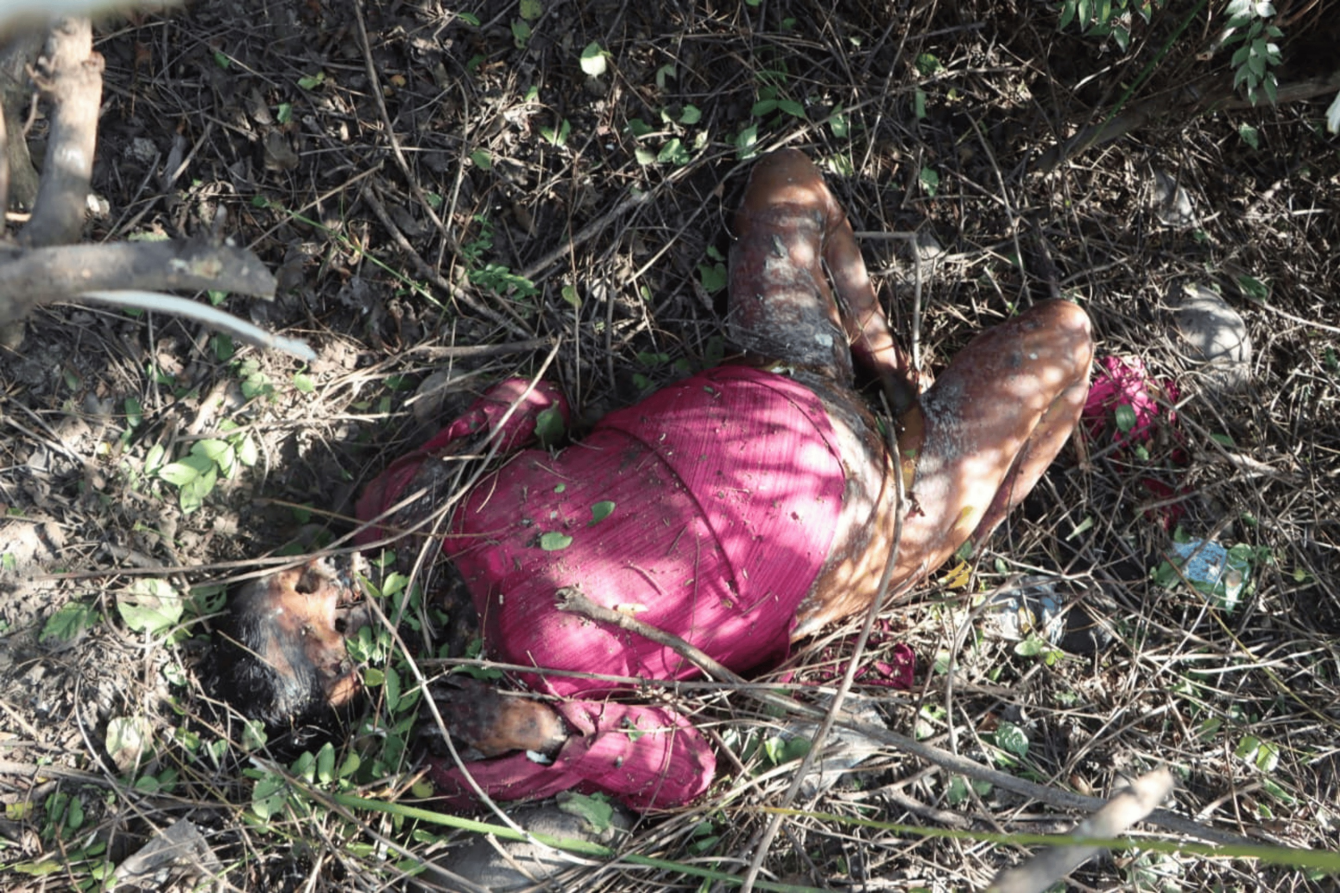
Body found in half-naked condition (Case 2) The body in Case 2 was discovered in a half-naked state in a vegetated area, raising suspicions of foul play.

**Figure 6:**
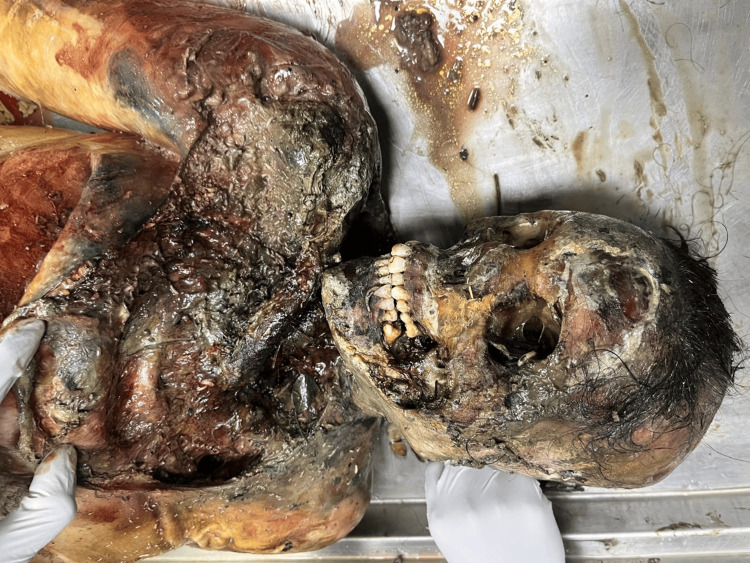
State of body received (Case 2) Severe decomposition in Case 2 with loss of soft tissue and signs of adipocere formation, typical of a moist environment.

**Figure 7:**
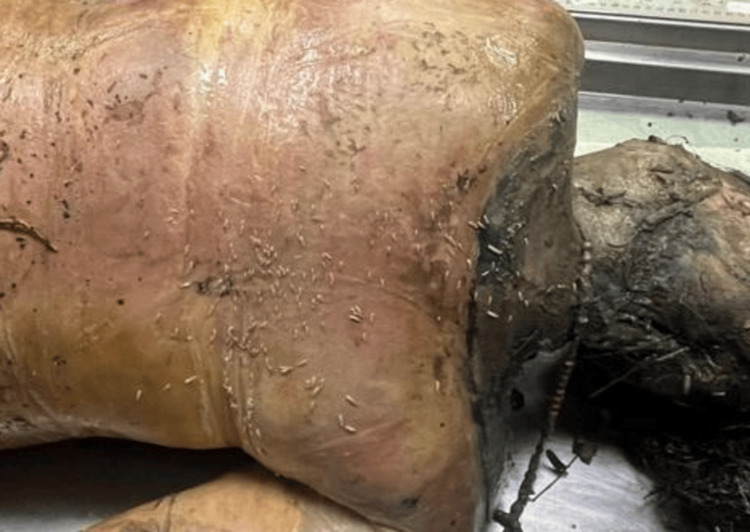
Maggots (Case 2) Larval infestation in Case 2, ranging from early instar to post-feeding stages, is indicative of a prolonged PMI. PMI: post-mortem interval

A 54-year-old female was discovered in a deserted colony plot with dense vegetation in winter season, 28 days after she was last seen. The body was reportedly found half-naked, raising concerns of possible foul play (Figure 5). Externally, there was extensive loss of skin and soft tissue over the face, jaw, neck, upper chest, and limbs, with clear signs of maggot and animal activity (Figure 6). Adipocere (a waxy, soap-like substance formed during decomposition in moist environments) had developed on parts of the body. Internally, the organs appeared greenish and soft, with honeycombing in the liver suggestive of prolonged putrefaction. Larval stages included early instars to post-feeding stages (Figure 7). Rainfall during the period of disappearance may have influenced the decomposition process and insect colonization patterns. Animal activity was observed.

GDD and ADH from the day of missing to the day of recovery of the body are depicted in Table 2.

**Table 2:** Case 2: GDD and ADH from the day of missing to the day of recovery of the body Case 2: Daily temperature data and corresponding GDD and ADH values used to estimate mPMI over a 28-day period. mPMI: minimum post-mortem interval; GDD: growing degree day; ADH: accumulated degree hours

Day	Avg temp	Threshold temp	GDD value (avg temp-threshold temp)	ADH (GDDx24 hrs)	Adding ADH from the day of recovery to the day of missing
D-0	13.5	10	3.5	84	2680
D-1	15.5	10	5.5	132	2596
D-2	15	10	5	120	2464
D-3	16	10	6	144	2344
D-4	13	10	3	72	2200
D-5	14.5	10	4.5	108	2128
D-6	15	10	5	120	2020
D-7	12.5	10	2.5	60	1900
D-8	13.5	10	3.5	84	1840
D-9	11	10	1	24	1756
D-10	12	10	2	48	1732
D-11	14	10	4	96	1684
D-12	14	10	4	96	1588
D-13	14	10	4	96	1492
D-14	13.5	10	3.5	84	1396
D-15	12.5	10	2.5	60	1312
D-16	14.5	10	4.5	108	1252
D-17	13	10	3	72	1144
D-18	12	10	2	48	1072
D-19	12.5	10	2.5	60	1024
D-20	13	10	3	72	964
D-21	14.5	10	4.5	108	892
D-22	15	10	5	120	784
D-23	12	10	2	48	664
D-24	15	10	5	120	616
D-25	14	10	4	96	496
D-26	15.5	10	5.5	132	400
D-27	17.5	10	7.5	180	268
D-28 (till 11 AM)	18	10	8	88	88

**Figure 8:**
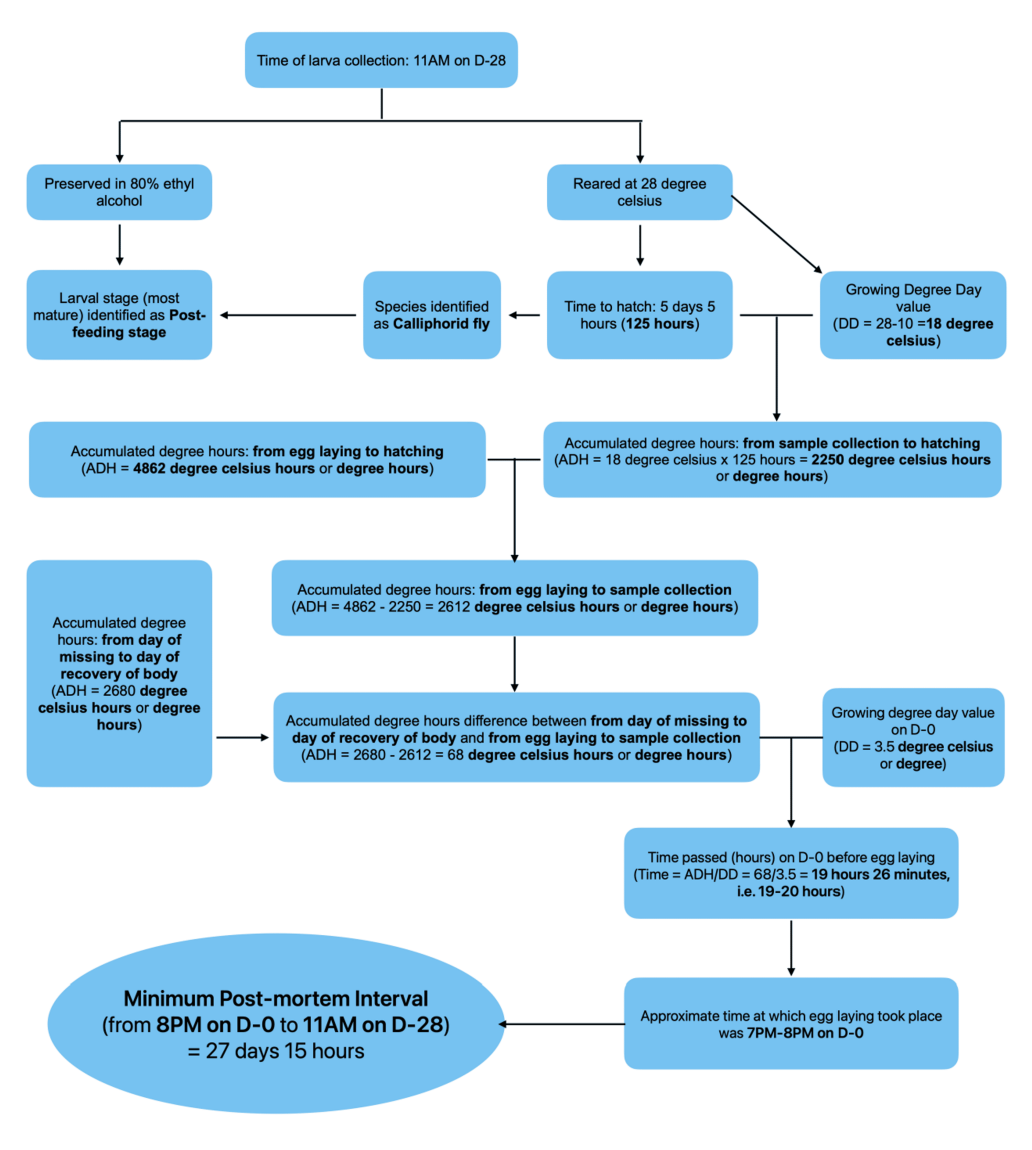
Flowchart: PMI estimation for Case 2 Flowchart detailing the process of PMI estimation for Case 2 based on ecological and entomological data.

mPMI estimated was about 27-28 days prior to post-mortem examination (Figure 8).

Case 3

**Figure 9:**
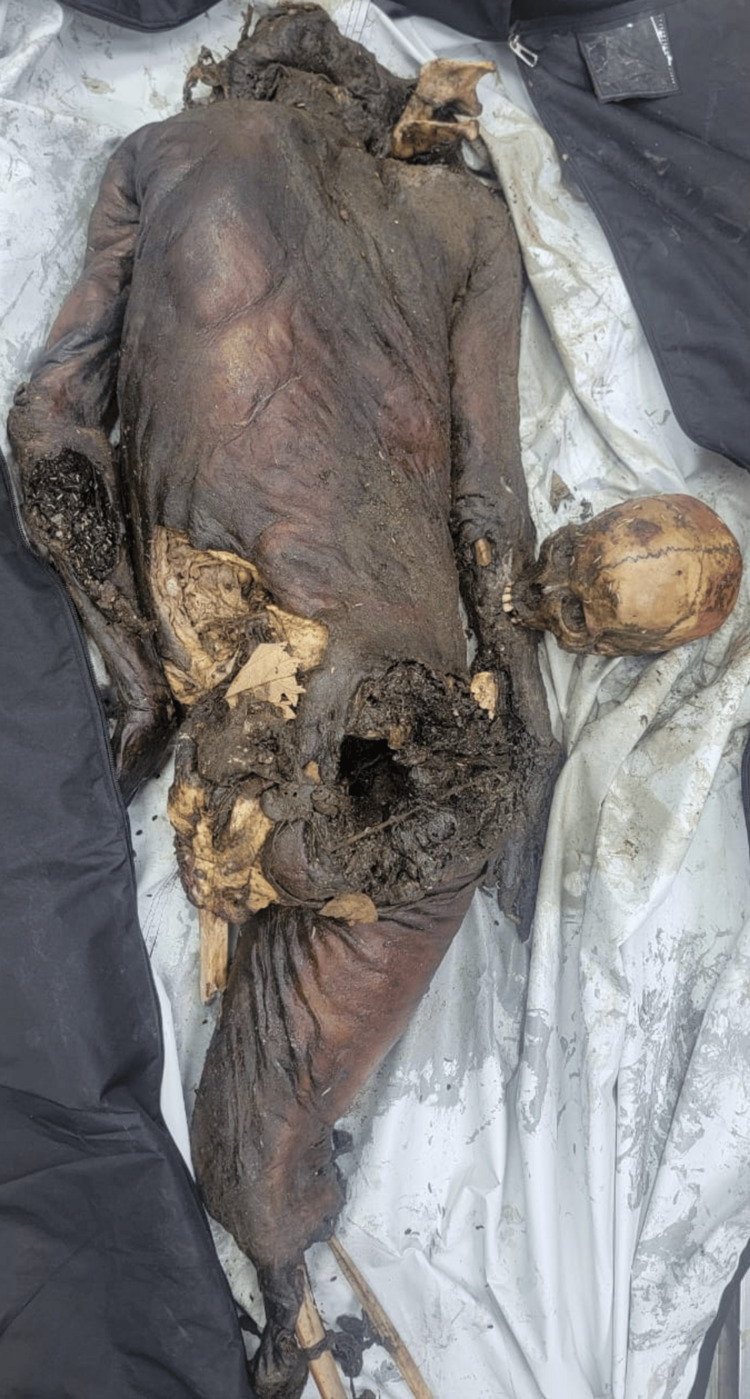
State of the body received (Case 3) Advanced decomposition of the body in Case 3, with the skeletal skull disarticulated, suggesting scavenger activity and prolonged exposure.

**Figure 10:**
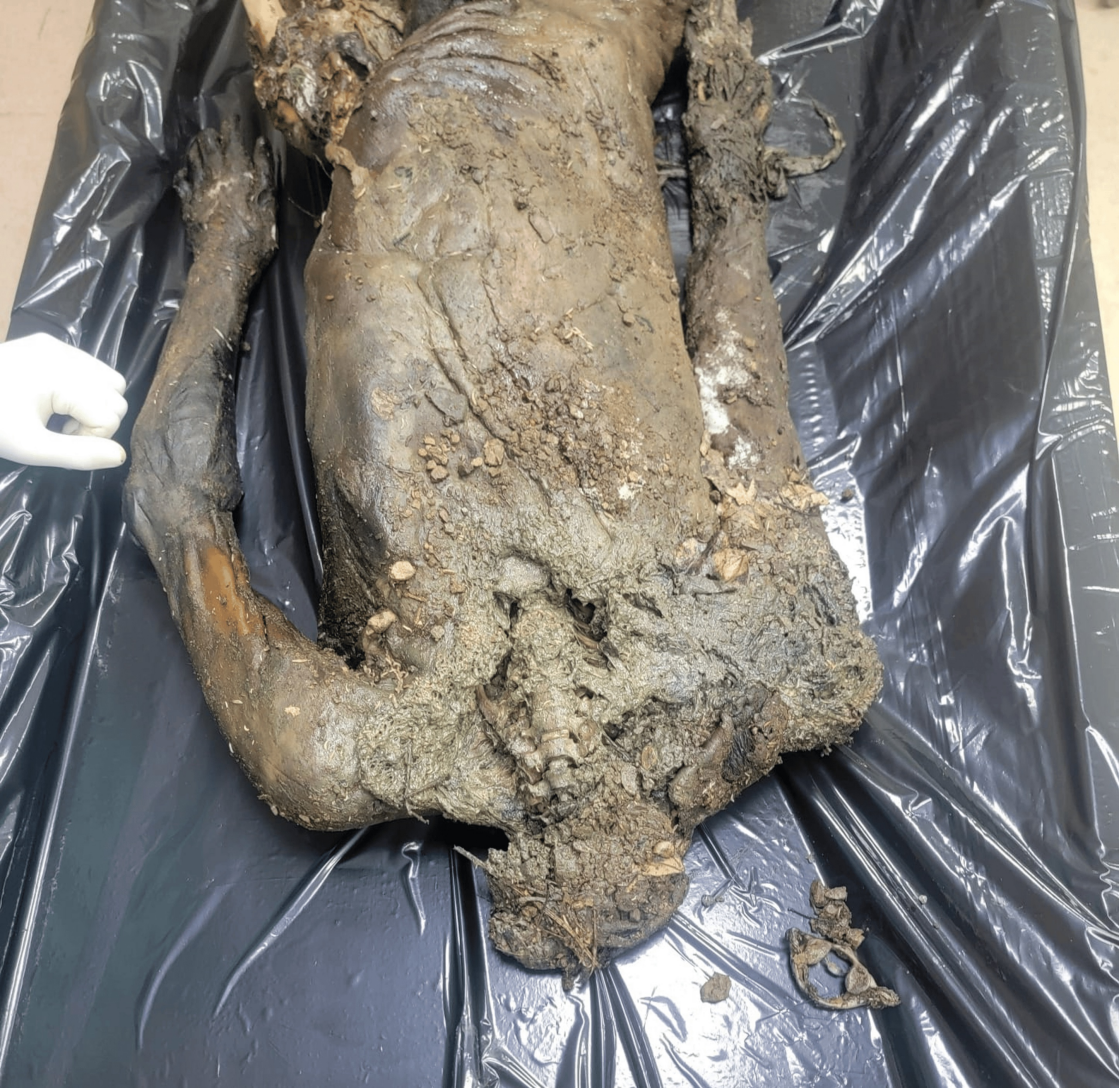
Skin absent over the neck and upper part of the chest (Case 3) The loss of skin over the neck and chest region in Case 3 was caused by insect and animal predation.

**Figure 11:**
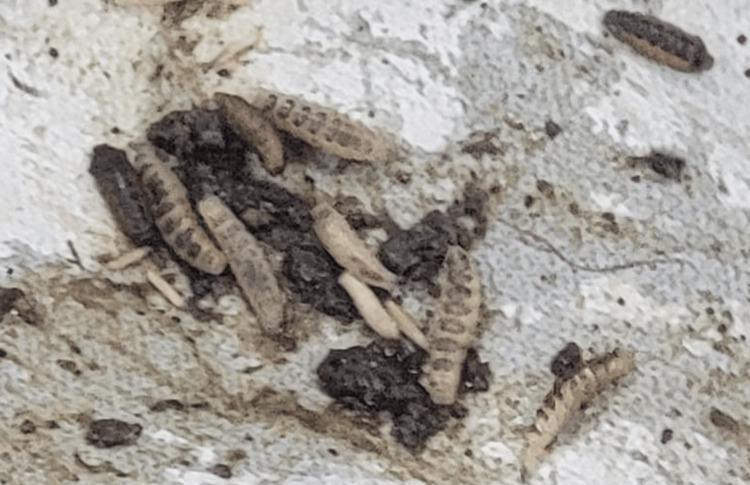
Maggots (Case 3) Multistage maggot colonization in Case 3, including pupae and adult Calliphoridae, indicating prolonged exposure.

A 17-year-old male was found dead in an open area on a hill, hidden among bushes during the spring season, 16 days after being reported missing. There was an alleged history of murder by a friend. The skull had become skeletonized and was separated from the body, indicating advanced decomposition and possible scavenger disturbance (Figure 9). Skin loss was noted over the neck, upper chest, and limbs, attributed to insect and animal activity (Figures 9, 10). The presence of maggots in all developmental stages, including pupae and adult flies (Calliphoridae), as well as beetles (Thanatophilus) and their larvae, suggested prolonged exposure (Figure 11). Internally, the thoracic organs were shrunken and discolored; the abdominal organs were soft, with a honeycombed liver. Animal activity was evident as part of the upper limbs and the left lower limb were absent.

GDD and ADH from the day of missing to the day of recovery of the body are depicted in Table 3.

**Table 3:** Case 3: GDD and ADH from the day of missing to the day of recovery of the body Case 3: GDD values and ADH indicating temperature-dependent insect development over a 16-day period. ADH: accumulated degree hours; GDD: growing degree day

Day	Avg temp	Threshold temp	GDD (avg temp-threshold temp)	AHD (GDDx24 hrs)	Adding ADH from the day of recovery to the day of missing
D-0	29.5	10	19.5	468	7094
D-1	29	10	19	456	6626
D-2	29	10	19	456	6170
D-3	27	10	17	408	5714
D-4	26.5	10	16.5	396	5306
D-5	27	10	17	408	4910
D-6	26.5	10	16.5	396	4502
D-7	25.5	10	15.5	372	4106
D-8	27	10	17	408	3734
D-9	28	10	18	432	3326
D-10	28	10	18	432	2894
D-11	28.5	10	18.5	444	2462
D-12	28	10	18	432	2018
D-13	27.5	10	17.5	420	1586
D-14	27	10	17	408	1166
D-15	27	10	17	408	758
D-16 (till 8 PM)	27.5	10	17.5	350	350

**Figure 12:**
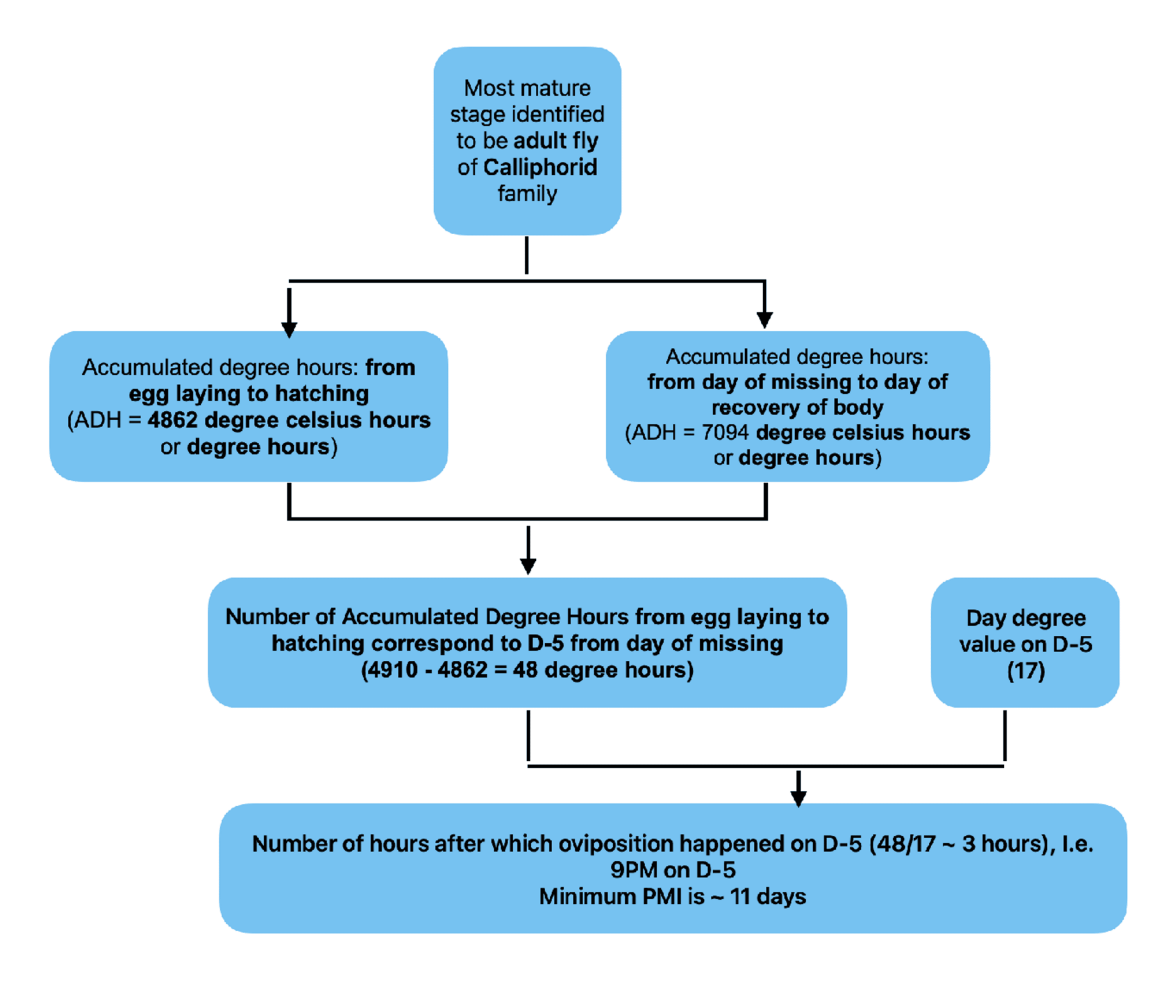
Flowchart: PMI estimation for Case 3 PMI estimation flowchart for Case 3 highlighting the entomological approach and thermal summation method. PMI: post-mortem interval

 mPMI estimated was about 11 days prior to post-mortem examination (Figure 12).

## Discussion

Estimating PMI based on physical and chemical changes after death is typically effective in the immediate to early PMI, that is, before the onset of putrefaction [1]. As the time since death increases, a more precise PMI can be derived using ecological data [4]. The association between the body development of insects and PMI is well established [3,5]. The common families of insects or flies colonizing the body are Calliphoridae, Sarcophagidae, and Muscidae, purely because of their size, number, and presence in all geographies. Different species of insects colonize the carrion at different stages of decomposition (Table 4) [6].

**Table 4:** Different species of insects colonize the carrion at different stages of decomposition Stages of decomposition and associated insect species colonizing human remains, with timeframes and characteristic observations for each stage. BOD: body organic debris

Stage	Timeframe	Key events and observations
Fresh stage	Days 1-2	Autolysis begins with minimal visible changes. Calliphorid flies (*Chrysomya megacephala, Chrysomya rufifacies*) arrive within 10 minutes, feeding on body fluids. Drosophilid species gather but do not lay eggs. *Diplonevra peregrina* flies appear in smaller numbers. By 6 hours, the staphylinid beetle (*Creophilus maxillosus*) is found beneath the carcass
Bloated stage	Days 2-7	Putrefaction begins, and gases inflate the abdomen, causing a balloon-like appearance. There is high Diptera activity, primarily by *C. megacephala* and *C. rufifacies*. By day 4, early instar larvae cover the head. Eggs of *Parasarcophaga misera* are laid by day 2. Predators such as *Nabis lusciosus*, *C. maxillosus*, and *Thinopinus albertisi* appear
Decay stage	Days 5-13	The carcass deflates as the abdominal wall is breached. Internal temperatures peak at 14°C above ambient. Strong decomposition odors develop, accompanied by rapid weight loss (20% of the original weight by day 10). Dominant organisms include third instar larvae of *C. megacephala* and *C. rufifacies*. Additionally, *Ophyra​​​​​​​* aenescens*, Ophyra​​​​​​​* chalcogaster*, Atherigona​​​​​​​* orientalis*,* and *Pollenia​​​​​​​* australis** are abundant
Post Decay Stage	Days 10-23	Large Diptera larvae leave, leaving behind bones, cartilage, hair, and decay by-products (BOD), which attract sphaerocerids (*Leptocera​​​​​​​* punctipennis*, Leptocera​​​​​​​* bifrons**), phorids (*Phora​​​​​​​ lucifera*), *P. australis*, and psychodids (*Psychoda*​​​​​​​* pseudoalternata*). Carcass weight stabilizes at 18%, with dominance by staphylinid beetles (*Paederus​​​​​​​ longicornis*, *T.* *albertisi*), dermapterans, and isopods
Remains Stage	Days 18-90+	Bones remain with little cartilage, and BOD becomes indistinguishable from soil. Diptera populations decline. Teneral psychodids are collected on day 26, though few persist afterward. Dermaptera and Isopoda populations increase initially, then decrease. Larval staphylinids disappear by day 30, with only a few adults remaining. Small but consistent ant populations are present

The PMI calculated based on the age of the largest immature stage was the mPMI, as adult flies seldom lay eggs on a live host, except in cases of myiasis. Furthermore, there is always a time gap between oviposition and the death of an individual [10]. Maximum PMI can be established in cases where the person’s last seen alive time is known, or by the succession method, which is based on data regarding the time between death and the appearance of arthropod species [11]. However, there are several factors that alter the colonization process viz., individual species characteristics, weather and seasonality, maggot mass effect, drugs and other toxins, geographic region, insect colonization preceding death, and presence of antemortem wounds [6].

To seek a solution to this issue, one should be aware of the life cycle and various factors related to body colonization by insects, oviposition, and their maturation from eggs to adult flies by traversing multiple immature stages. Insect development depends on specific temperature ranges (upper and lower developmental thresholds). These thresholds vary according to the species and life stages. Insects require these temperature ranges to accumulate sufficient heat to advance from oviposition to hatching. This "thermal summation" approach uses ADH or ADD to measure thermal time. This measurement applies to each developmental stage (egg, instar, and pupa), with each stage requiring a specific amount of ADH or ADD [3].

Different developmental stages of calliphorid flies require different amounts of ADH to mature, which when reared at a fixed temperature (23°C) provides the data in hours (Table 5) [10].

**Table 5:** Time taken by a Calliphorid fly to develop through its different stages Development time (in hours) of different life stages of a Calliphorid fly at a constant temperature of 23°C, used for PMI estimation.

Development stage	Development time (hrs) at 23°C
Egg	19
1st instar larvae	18
2nd instar larvae	33
3rd instar larvae	44
Post-feeding	92
Pupae	168
Egg to adult	374

From the above-mentioned table, GDD and ADH can be calculated (Table 6).

**Table 6:** Calculation of ADH for each developmental stage at 23°C Calculation of GDD values and corresponding ADH required for each developmental stage of the Calliphorid fly at 23°C (The lower developmental threshold for the Calliphorid fly is 10°C; at 23°C, GDD=23−10=13°C). ADH: accumulated degree hours; GDD: growing degree day

Developmental stage	GDD value (avg temp-threshold temp)	Time in hrs	ADH (GDDxtime in hrs.)	Total ADH
Egg	13	19	247	247
1st instar larvae	13	18	234	481
2nd instar larvae	13	33	429	910
3rd instar larvae	13	44	572	1482
Post-feeding	13	92	1196	2678
Pupae	13	168	2184	4862

**Figure 13:**
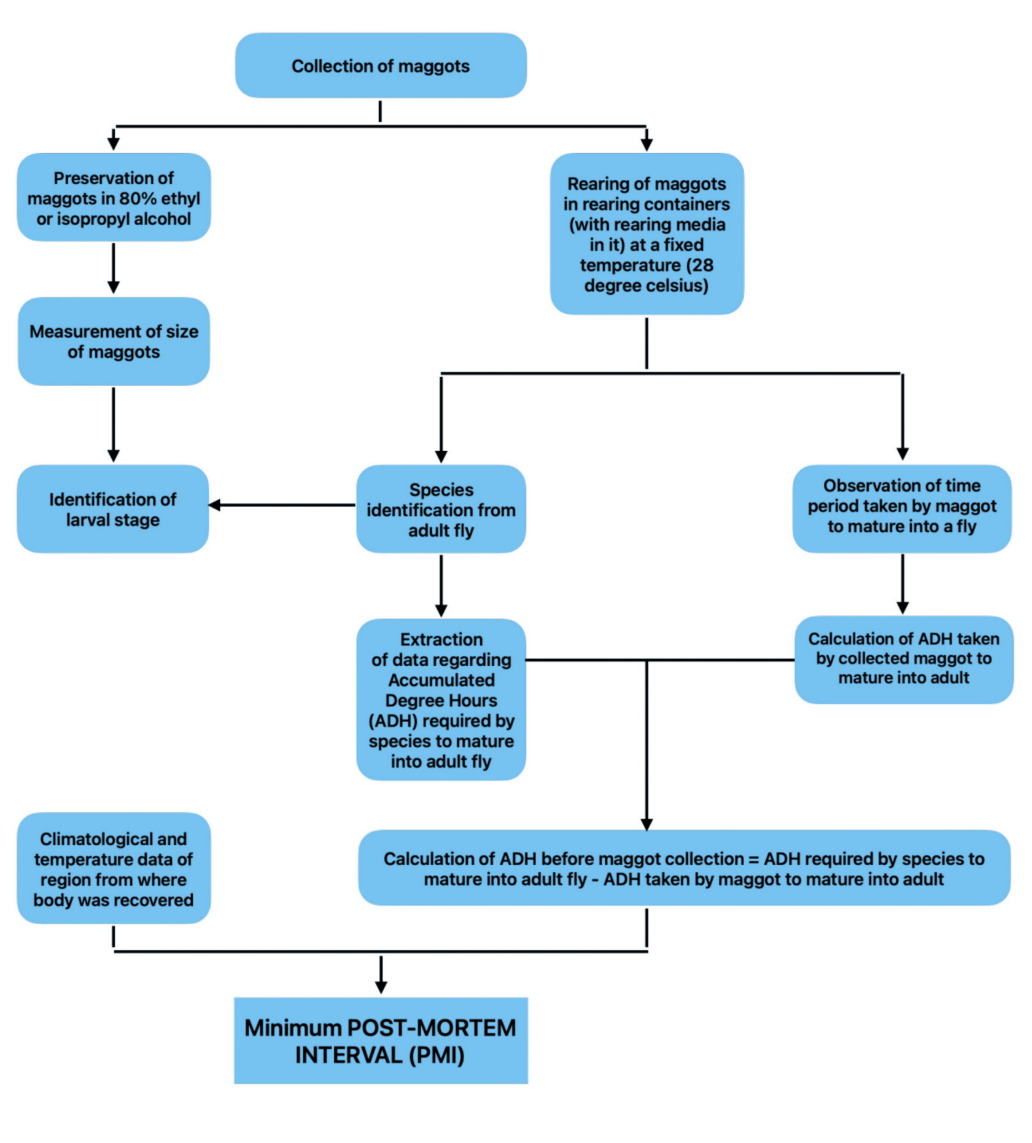
Methodology for PMI estimation using entomology Stepwise methodology for PMI estimation using forensic entomology including collection, rearing, species identification, and calculation based on ADH. ADH: accumulated degree hours; PMI: post-mortem interval

Above mentioned data in Tables 5, 6 can be used in the estimation of PMI by using a simple process depicted in Figure 13.

In our cases, we have used the Calliphorid fly as entomological evidence to estimate PMI, as it is one of the earliest insects to colonize a carrion. Calliphorid fly can be used to estimate longer PMIs as compared to conventional methods currently in practice. One of the major drawbacks of estimating even longer PMIs can be overcome by the use of other insect species, which appear at a later stage during the decomposition process. This warrants further research on different insect species to derive simplified methods for the estimation of PMI.

## Conclusions

Based on our case series, forensic entomology is an essential tool in criminal investigations, particularly for estimating the PMI in cases of advanced decomposition where traditional methods fall short. The presented cases underscore how entomological evidence, especially Calliphorid fly development, effectively provides PMI estimates even in the presence of significant tissue damage across various environments and seasons.

While precise, the estimation of PMI through insect development is influenced by numerous factors, including environmental conditions, insect behavior, and host-specific variables. Our detailed methodology, incorporating controlled rearing of collected larvae, precise species identification, thermal summation using ADH, and the integration of local climatological data, allows for a structured approach to navigate these complexities and determine the mPMI. When integrated with other investigative data, this entomological approach significantly refines the time-of-death estimate, providing crucial, impartial scientific insights and filling vital investigative gaps. Continued research into the ecology and life cycle of diverse local insect species will further enhance the precision and indispensable contribution of forensic entomology to forensic medicine.
